# The International Medical Congress

**Published:** 1881-08-20

**Authors:** 


### *THE INTERNATIONAL MEDICAL CONGRESS*

Assembled in London on the third day of August, 1881. The meeting was opened by an address from the Prince of Wales, and the inaugural address was made by the President, Sir James Paget.

Three thousand members were present as representatives of the profession from all parts of the world.

There were present Sir Wm. Jenner, Sir Wm. Gull, Mr. McCormack, the Crown Prince of Prussia, and other dignitaries. Thus giving patronage and honor to the profession of medicine never, perhaps, before extended in so marked a degree.

